# Tracking Genetic Parkinson's Disease with Molecular Imaging: A Systematic Review

**DOI:** 10.1002/mdc3.70687

**Published:** 2026-06-04

**Authors:** Chiara Meneghini, Luca Gallo, Arianna Sala, Enza Maria Valente, Micol Avenali, Silvia Paola Caminiti

**Affiliations:** ^1^ Department of Brain and Behavioral Sciences University of Pavia Pavia Italy; ^2^ Coma Science Group, GIGA Consciousness, GIGA Institute, University of Liège Liège Belgium; ^3^ NeuroRecovery Lab, GIGA Consciousness, GIGA Institute, University of Liège Liège Belgium; ^4^ NeuroRehab & Consciousness Clinic, Neurology Department University Hospital of Liège Liège Belgium; ^5^ IRCCS Mondino Foundation Pavia Italy; ^6^ Department of Molecular Medicine University of Pavia Pavia Italy

**Keywords:** precision medicine, biomarker, neuroimaging, nuclear medicine, parkinsonism

## Abstract

**Background:**

Parkinson's disease (PD) is a worldwide, complex neurodegenerative disorder influenced by both genetic and environmental factors. Around 15–20% of PD cases are linked to genetic mutations, providing insights into the disease's pathogenesis.

**Objective:**

The current review aims to summarize molecular neuroimaging findings in genetic forms of PD, also considering the pre‐symptomatic phase of the disease to identify markers distinguishing genetic PD from sporadic forms of the disease as well as early markers of disease conversion.

**Methods:**

A literature search on PubMed and Scopus was performed to identify molecular imaging studies on symptomatic and asymptomatic carriers of the following PD‐related mutations: *SNCA*, *PRKN*, *PINK1*, *PARK7*, *LRRK2* and *GBA1*.

**Results:**

A total of 96 studies were summarized to highlight mutation‐specific brain alterations, considering dopaminergic and extra‐dopaminergic systems (serotonin, acetylcholine), metabolic and cerebral blood flow alterations as well as emerging evidence on beta‐amyloid, tau proteins and neuroinflammation.

**Conclusions:**

Molecular neuroimaging techniques, including Positron Emission Tomography (PET) and Single Photon Emission computed tomography (SPECT), provide valuable tools for tracking PD pathophysiology in vivo. Indeed, nuclear imaging is able to add information on genetic PD, offering possible biomarkers to stratify patients, predict cognitive decline, and support personalized therapeutic approaches.

Parkinson's Disease (PD) is a progressive neurodegenerative disorder, characterized by motor symptoms—bradykinesia, resting tremor, rigidity—and non‐motor manifestations, complicating disease management.^1^, ^2^, ^3^ Aging is the main risk factor associated with PD, and at the time of diagnosis, at least half of the substantia nigra (SN) dopaminergic projections to the striatum are already lost.^4^, ^5^, ^6^, ^7^

Historically considered a sporadic age‐related disorder, PD manifestation is determined by a combination of environmental and genetic risk factors.^8^ The discovery of mutations in the SNCA gene changed our understanding of PD by revealing a direct genetic cause and identifying alpha‐synuclein (α‐syn) aggregation as a central mechanism in its pathogenesis.^5^, ^9^ To date, pathogenic variants in 22 distinct genes have been linked to PD, including autosomal dominant and recessive forms (*PRKN*, *PINK1, SNCA, PARK7, VPS35)* and strong genetic risk factors (such as *LRKK2*, *GBA1 and RAB32)*.^8^, ^10^, ^11^

Heterozygous missense variants in the *SNCA* gene cause a rare dominant form of PD.^12^, ^13^, ^14^, ^15^ SNCA‐associated neurodegeneration likely involves a toxic gain‐of‐function mechanism where impaired α‐syn processing triggers pathological aggregation, Lewy body (LB) formation, reactive oxygen species (ROS) and energy depletion.^12^ SNCA‐PD is characterized by young onset, fast disease course, rapid cognitive decline, dysautonomia, hallucinations, delusions, myoclonus, and pyramidal symptoms (Fig. 1A).^12^, ^13^, ^14^, ^15^

**Figure 1:**
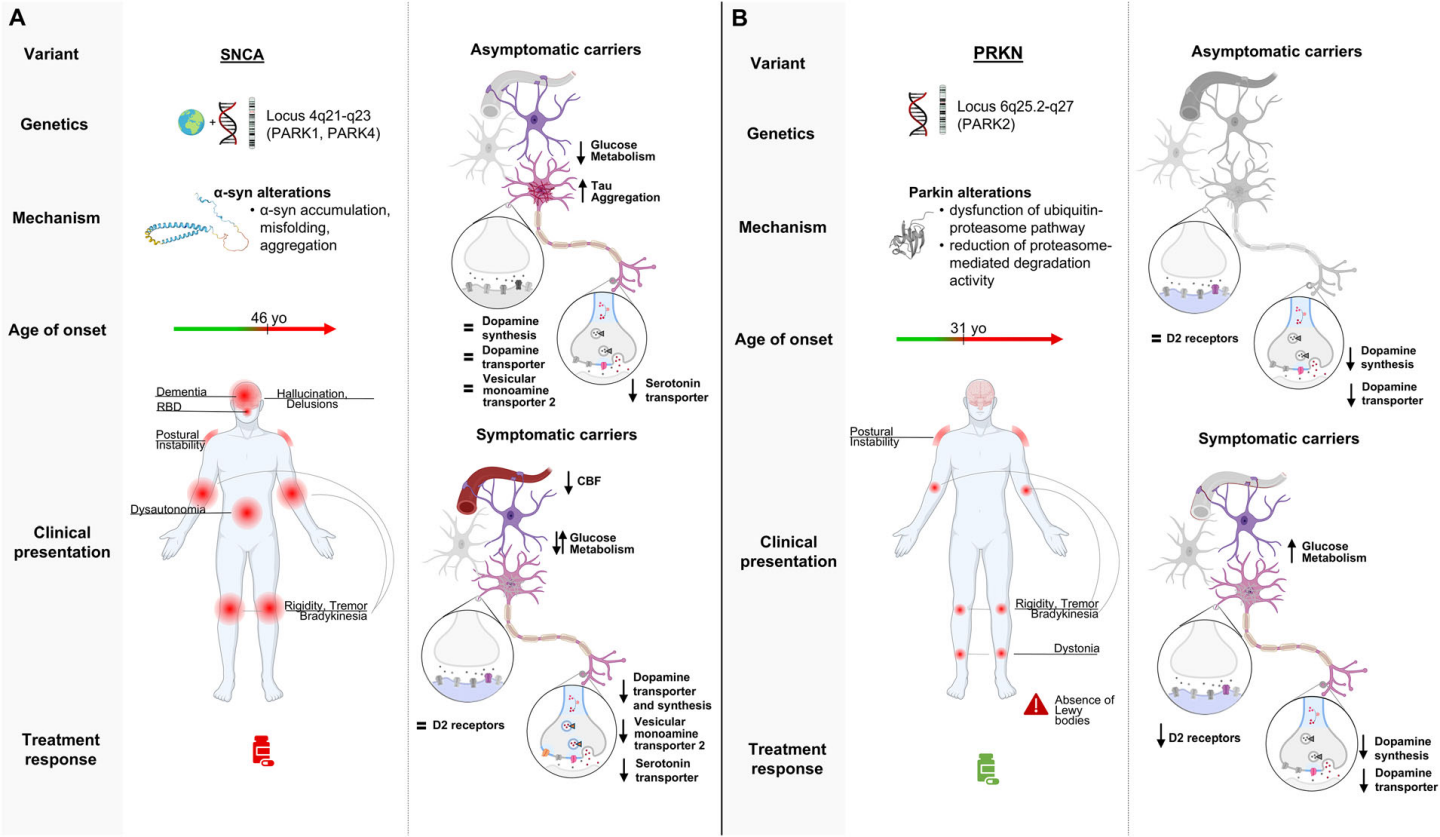
Clinical and neuroimaging features of *SNCA* and PRKN symptomatic and asymptomatic carriers. Pathological changes, clinical and neuroimaging features, pharmacological treatment responses and known alterations in molecular imaging in (A) *SNCA* and (B) PRKN mutations carriers. Figure was partially created using BioRender (https://biorender.com/). alpha‐synuclein (α‐syn); cerebral blood flow (CBF); REM sleep behavior disorder (RBD); years old (yo).

Biallelic variants in the *PRKN* gene (*PARK2*) are the most common cause of autosomal recessive early‐onset PD. *PRKN* mutations impair the ubiquitin‐proteasome clearing system, causing accumulation of misfolded protein, damaged organelles, and accelerated neuronal death.^16^ PRKN‐PD appears before the age of 40, lacks LBs formation, exhibits slow progression, good and sustained levodopa response, symmetric motor impairment, variable occurrence of dystonia at onset, hyperreflexia, diurnal fluctuations, and sleep benefit.^17^, ^18^, ^19^ Patients have a higher risk of early motor complications (Fig. 1B).^18^

Mutations in the *PINK1* gene (*PARK6*) cause autosomal recessive PD.^20^ PINK1‐related neurodegeneration arises from a loss‐of‐function mechanism leading to mitochondrial dysfunction.^21^, ^22^ As for Parkin, *PINK1*‐*PD* is characterized by early onset, slow disease progression, and excellent levodopa response, contributing to a relatively favorable long‐term prognosis (Fig. 2A).^23^, ^24^

**Figure 2:**
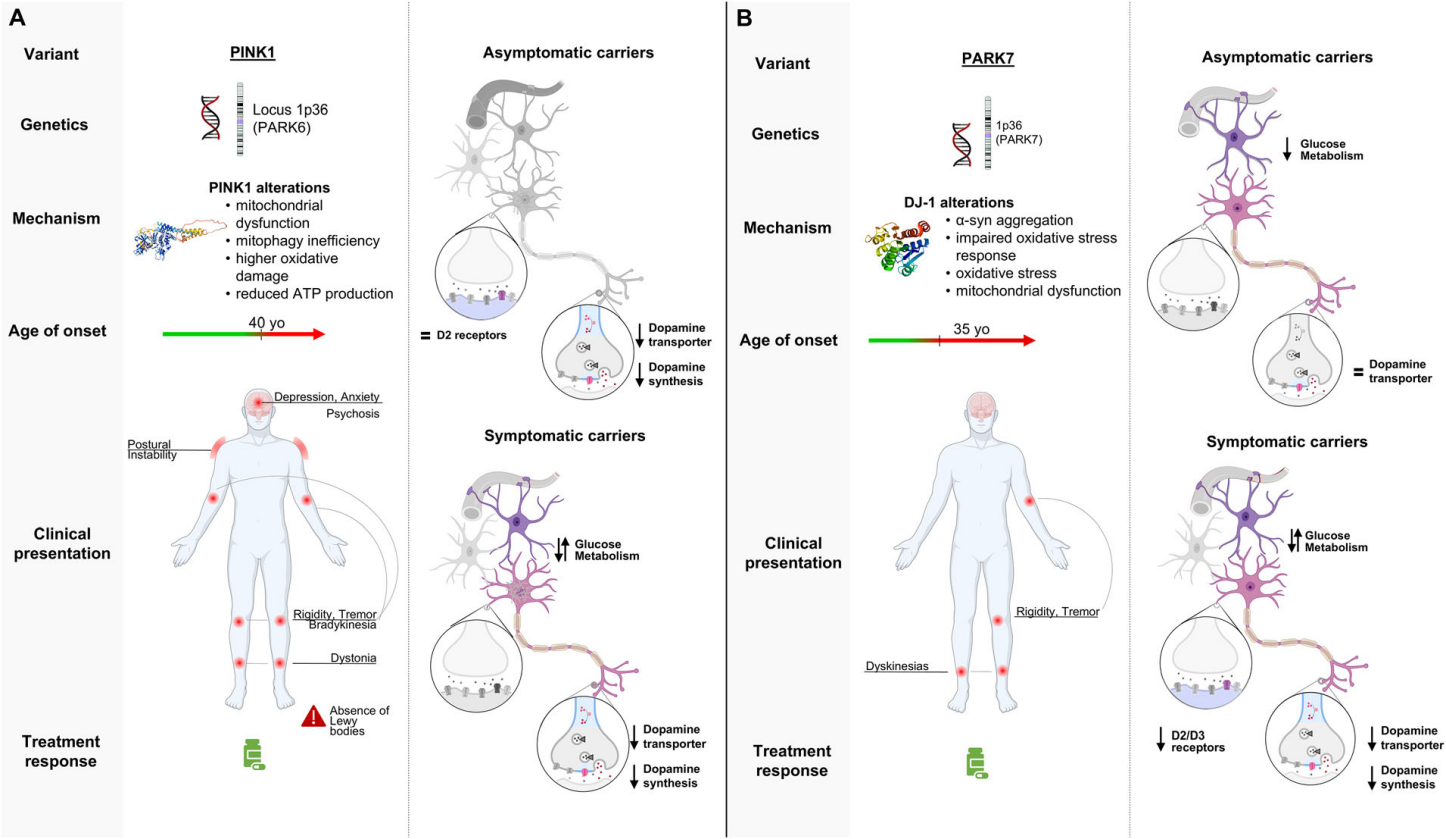
Clinical and neuroimaging features of *PINK1* and *PARK7* symptomatic and asymptomatic carriers. Pathological changes, clinical and neuroimaging features, pharmacological treatment responses and known alterations in molecular imaging in (A) *PINK1* and (B) *PARK7* mutations carriers. Figure was partially created using BioRender (https://biorender.com/). years old (yo).

*PARK7* (encoding the DJ‐1 protein) causes autosomal recessive early‐onset PD.^25^, ^26^, ^27^ DJ‐1 is a redox‐sensitive chaperone contrasting ROS and inhibiting α‐syn aggregation, preventing toxic protein formation.^25^, ^26^, ^27^ Similar to other recessive forms, *PARK7*‐PD exhibits slow progression, good levodopa response with drug‐induced dyskinesias (Fig. 2B).^25^, ^26^, ^27^, ^28^

Mutations of the *VPS35* gene were associated with rare autosomal dominant PD. VPS35 is crucial for the normal functioning of the multimeric retromer complex, which mediates endosome trafficking. Thus, *VPS35* mutations lead to α‐syn and ROS accumulation.^29^, ^30^, ^31^ Clinically, *VPS35‐PD* resembles sPD, with good levodopa response.^32^, ^33^

Heterozygous variants in the *LRRK2* gene (*PARK8*) represent one of the most common and strong genetic risk factors for PD.^34^ The LRRK2‐encoded kinase—dardarin—is involved in the phosphorylation of proteins (α‐syn and microtubule‐associated tau).^35^, ^36^, ^37^ Post mortem studies revealed heterogeneous pathological alterations in *LRRK2* carriers involving LBs, pure nigral degeneration, diffuse LB disease, neurofibrillary tangles and tau‐related pathologies.^38^, ^39^, ^40^ This suggests that *LRRK2* mutations may contribute to an array of neurodegenerative processes.^38^, ^39^, ^40^ *LRRK2*‐*PD* resembles idiopathic PD, with a mean age of onset of 65, unilateral tremor, good levodopa response and slow disease progression.^36^ Cognitive decline is usually absent in early stages, contributing to a benign disease course (Fig. 3A **)**.^36^

**Figure 3:**
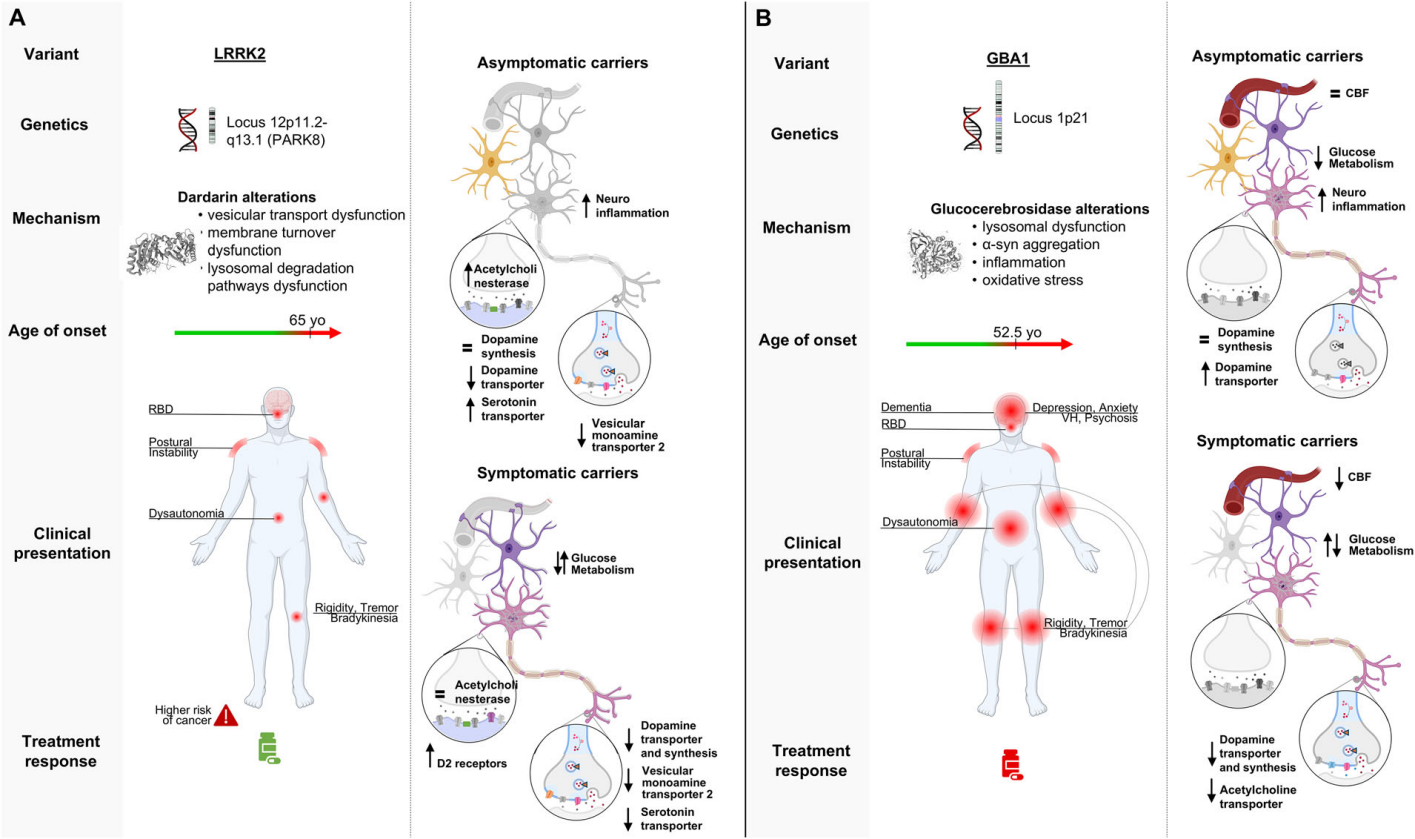
Clinical and neuroimaging features of *LRRK2* and *GBA1* symptomatic and asymptomatic carriers. Pathological changes, clinical and neuroimaging features, pharmacological treatment responses and known alterations in molecular imaging in (A) *LRRK2* and *GBA1* (B) mutations carriers. Figure was partially created using BioRender (https://biorender.com/). cerebral blood flow (CBF); REM sleep behavior disorder (RBD); visual hallucinations (VH); years old (yo).

Heterozygous variants of the *GBA1* gene—the commonest genetic PD risk factor worldwide—result in reduced activity of glucocerebrosidase (GCase) enzyme, leading to glucosylceramide build‐up and autophagic‐lysosomal pathway disruption.^41^, ^42^, ^43^ Reduced GCase activity leads to α‐syn accumulation, which further impairs GCase trafficking to the lysosome, exacerbating PD underlying pathology.^44^, ^45^ *GBA‐PD* patients exhibit a younger age of onset with comparable clinical presentation to sporadic cases, but a higher occurrence of non‐motor features and faster progression of cognitive symptoms (Fig. 3B).^46^, ^47^

Finally, mutations of the *RAB32* gene are the newest discovered PD genetic risk factor. The RAB family involves 61 GTPases that function as molecular switches to regulate intracellular vesicular trafficking. Mutations of *RAB32* lead both to an hyperactivation of LRRK2 kinase and lower colocalization with PINK1.^48^ Further, *RAB32‐PD* in‐depth clinical characterizations are needed.

## Molecular Imaging in Sporadic PD: Context for Genetic Studies

Molecular neuroimaging techniques, including positron emission tomography (PET) and single photon emission computed tomography (SPECT), provide valuable tools to investigate molecular mechanisms underlying neurodegenerative conditions. These techniques demonstrated accuracy in supporting early and differential PD diagnosis and tracking PD pathology in‐vivo.

[123I]‐FP‐CIT is the most used radiotracer to measure dopamine transporter (DAT) activity.^49^, ^50^, ^51^ DAT imaging, via SPECT, represents a supportive biomarker for PD.^3^ SPECT striatal uptake correlates with motor symptoms severity and captures dopaminergic deficits in 50% of subjects with rapid eye movement (REM) sleep behavior disorder (RBD), a prodromal condition for PD, suggesting it can track disease progression from its early stages.^52^, ^53^, ^54^, ^55^, ^56^ Other tracers investigating dopaminergic integrity include: [11C]TBZ, [11C]DTBZ and [18F]FP‐DTBZ for Vesicular Monoamine Transporter Protein 2 (VMAT2) density; [11C]Raclopride, [11C]NPA, [11C]MNPA and [11C]PHNO for extracellular dopamine levels.^57^, ^58^, ^59^ Dopaminergic integrity can be investigated in‐vivo also through PET.

Other neurotransmitter systems are gaining attention in PD. Serotonin dysfunction precedes dopaminergic denervation and motor symptoms.^7^ Reductions of [11C]DASB (serotonin transporter radiotracer, SERT) binding are observed in PD patients, from early disease stages.^60^, ^61^, ^62^ Decreased serotonin uptake is associated with tremors, fatigue and apathy, whereas increased SERT binding is linked with severe depressive symptomatology.^62^, ^63^, ^64^, ^65^, ^66^, ^67^ The concomitant SERT upregulation, typical of depression, and serotonin denervation, typical of PD, might explain these findings.^68^

Cholinergic denervation, assessed with [11C]MP4A and [11C]PMP—proxies of acetylcholinesterase activity, is prominent in PD with dementia.^69^, ^70^, ^71^, ^72^ Cholinergic system alterations can be detected from early disease phases in patients at high risk of developing dementia.^70^, ^73^

SPECT perfusion imaging reveals fronto‐parietal perfusion deficits in PD symptomatology, which differ from perfusion patterns exhibited by other neurodegenerative conditions.^74^, ^75^, ^76^ [18F]FDG radiotracer mirrors glucose metabolic consumption and represents a proxy of synaptic dysfunction.^76^, ^77^, ^78^ [18F]FDG‐PET analysis identifies two different patterns of metabolic alterations: PD‐related pattern (PDRP) and PD‐related cognitive pattern (PDCP). The first is characterized by hypermetabolism within pallidum, thalamus and pons and hypometabolism in supplementary motor area (SMA), premotor cortex, and parietal associative areas, correlating with motor and dopaminergic dysfunction.^79^, ^80^, ^81^, ^82^ The latter is described by hypometabolism within fronto‐parietal regions and hypermetabolism in the cerebellum and dentate nuclei, which correlated with cognitive decline/dementia.^79^, ^83^, ^84^, ^85^

Moreover, [11C]PiB positivity, an amyloid‐beta tracer, has been associated with deficits in global cognition, executive function, and language, predicting cognitive decline in PD.^86^

Recently, radiotracers ([18F]PI‐2620, [18F]AV‐1451) detecting in‐vivo tau pathology have shown that neocortical tau burden correlates with cognitive impairment (from mild to severe) in PD, proposing Tau‐PET as a promising marker of cognitive decline.^87^, ^88^, ^89^, ^90^

Other markers of disease pathology, such as neuroinflammation, are receiving growing attention. Indeed, post‐mortem and animal studies demonstrate activated microglia in PD.^91^

Moreover, novel tracers ([18F]‐C05‐05, [18F]ACI‐12589 and [18F]F0502B) targeting α‐syn pathology have been developed. [18F]ACI‐12589 demonstrated good affinity and specificity for pathological α‐syn aggregates and demonstrated binding in cerebellar white matter and middle cerebellar peduncles of multisystem atrophy patients, but not in PD.^92^ [18F]C05‐05, instead, was also able to capture α‐syn pathology in the midbrain of PD patients.^93^ [18F]F0502B can specifically recognize α‐syn fibrils both in‐vitro and in‐vivo, however, its applicability in human patients has yet to be tested.^94^ However, as of today, no α‐syn tracer has been extensively validated, thus molecular imaging targeting brain metabolism and cerebral blood flow (perfusion), neurotransmitters, and protein aggregates are the most established tools to investigate PD‐related brain changes.

Emerging studies reported differences between PD patients carrying genetic mutations and those with sporadic PD (sPD), with some alterations observable even in the preclinical stages. This review will focus on the application of nuclear imaging techniques in both genetic forms of PD and non‐manifesting mutation carriers (NMCs) with the aim of summarizing current knowledge on brain signatures that may distinguish genetic PD from sPD. By considering NMCs, we also aim to explore potential predictive biomarkers of phenoconversion. Indeed, NMCs offer a unique window into neuropathological changes preceding overt disease onset, enabling investigation of early pathogenic processes otherwise difficult to capture in sPD. In this context, molecular imaging provides the opportunity to detect in‐vivo subclinical changes in brain metabolism, neurotransmitter systems, and protein aggregations.

## Methods

### Search Strategy

A systematic search was conducted in accordance with PRISMA guidelines across two databases, Scopus (via Elsevier) and PubMed, up to April 2025. The search strategy included the following keywords: [1] ((mutation) OR (familial) (SNCA) OR (GBA) OR (LRRK2) OR (PRKN) OR (PINK1) OR (PARK2) OR (PARK7) OR (PARK1) OR (juvenile)) AND ((PET) OR (SPECT) OR (neuroimaging) AND (Parkinson)) [2] ((mutation) OR (familial) OR (SNCA) OR (GBA1) OR (LRRK2) OR (PRKN) OR (PINK1) OR (PARK2) OR (PARK7) OR (PARK1) OR (juvenile)) AND ((PET) OR (“positron emission tomography”) OR (SPECT) OR (neuroimaging)) AND (Parkinson). On Scopus database, the search was focused on original articles and letters only. The protocol of this review was not registered.

Due to the heterogeneity of the populations of interest, imaging modalities, outcome measures, and the inclusion of case reports and small cohort studies, a meta‐analysis was not feasible. Therefore, findings were synthesized using a narrative approach. Similarly, risk of bias assessment was not performed, as it would not have provided meaningful or comparable evaluations across studies.

### Inclusion and Exclusion Criteria

We included studies from March 1998 till April 2025. Studies not including genetic PD cohorts and PET/SPECT evaluations were excluded. *VPS35* and *RAB32* mutations were excluded from the search due to the low number of available findings. Review papers, conference abstracts, editorials and non‐English articles were excluded as well.

Two investigators (C.M and L.G.) independently evaluated abstracts and titles to remove non‐pertinent papers (Cohen's *k* = 0.67). Then, two other investigators (S.P.C. and A.S) screened the full text of the remaining articles to identify eligible ones (Cohen's *k* = 0.78).

## Results

After duplicates were removed, a total of 8560 articles were obtained. After title and abstract screening, 230 references were selected for full‐text reading. Of these, a total of 96 research papers were included in this review. Studies were mainly excluded due to irrelevant pathologies or absence of genetically determined PD cohorts (Fig. 4). All molecular findings of the included articles were considered and reported either in the main text or tables, or both. Molecular imaging findings were grouped based on genetic mutation, separating symptomatic and asymptomatic carriers. Findings were further divided based on the target of the used radioligand: dopaminergic system, other neurotransmitter systems (serotonin and acetylcholine mainly), proxy of brain activity (metabolism or perfusion studies), and proxy of brain pathology when present (beta‐amyloid and tau pathology as well as neuroinflammation).

**Figure 4:**
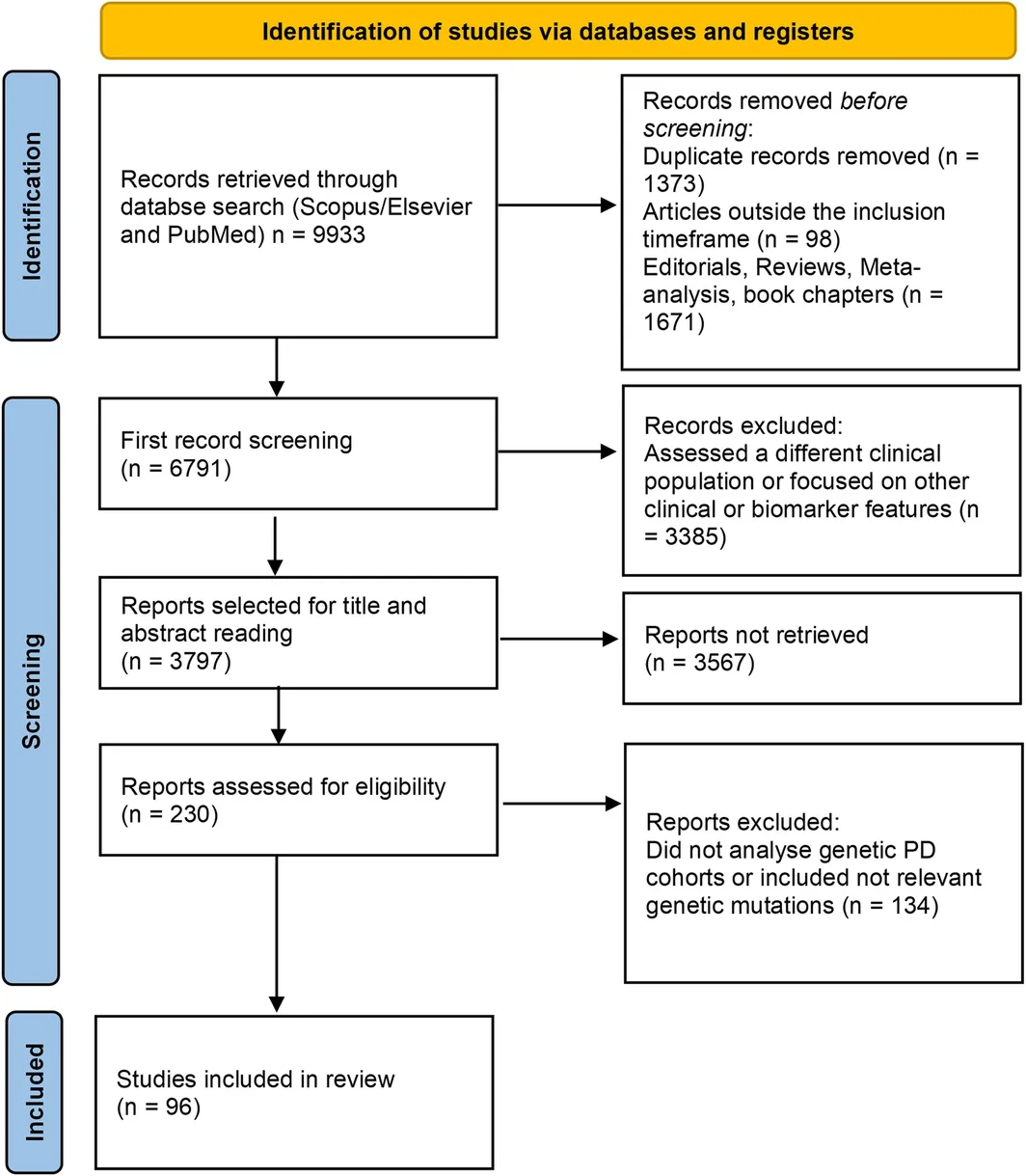
PRISMA flowchart diagram.

### Nuclear Imaging Findings in Genetic Subtypes of Parkinson's Disease

#### SNCA

A comprehensive assessment of dopaminergic damage in symptomatic and asymptomatic *SNCA* carriers is available, although findings vary depending on the specific mutation. Overall, *SNCA* manifesting carriers (MCs) exhibit reduced DAT and postsynaptic binding compared to sPD and non‐manifesting carriers (NMCs).^95^

Across variants, both symmetrical and asymmetrical patterns of degeneration have been described. For instance, G209A and A53T carriers typically show relatively symmetrical striatal dopaminergic loss, resembling sPD, with preserved D2 receptor binding.^96^, ^97^, ^98^, ^99^, ^100^, ^101^, ^102^ In contrast, other studies reported more extensive caudate involvement in A53T carriers, suggesting a heightened striatal vulnerability.^99^, ^103^ Moreover, A30P mutations have been associated with combined pre‐ and post‐synaptic dysfunction,^104^ while SNCA triplications and E46K variants tend to exhibit asymmetrical presynaptic degeneration.^15^, ^105^, ^106^, ^107^ On the other hand, *SNCA A53E‐MCs* [123I]FP‐CIT‐SPECT evaluation revealed symmetric putaminal dopaminergic degeneration.^108^

Controversial findings were obtained in NMCs, with some subjects displaying striatal integrity and others exhibiting degeneration of caudate and putamen.^15^, ^97^, ^98^, ^100^, ^102^, ^103^, ^104^, ^107^ This variability is consistent with the incomplete penetrance of SNCA missense mutations and gene duplications and suggests that additional genetic or environmental modifiers contribute to disease expression.^13^, ^95^, ^97^, ^103^, ^104^, ^107^ Notably, increased D2 receptor binding has been observed in A53T‐NMCs, potentially reflecting early compensatory mechanisms.^102^

Serotonergic dysfunction appears to be an early and prominent feature of SNCA carriers. Indeed, decreased SERT binding ([11C]DASB) was observed in multiple regions, including putamen, caudate, hypothalamus, amygdala and insula of *SNCA A53T‐MCs* compared to sPD.^103^ Similar serotonergic deficits have been identified in A53T‐NMCs despite preserved dopaminergic integrity, and their severity was comparable to sPD serotonergic damage.^103^ These findings align with experimental evidence indicating that SNCA mutations negatively regulate the availability of SERT on the cell surface.^109^ Moreover, brainstem SERT reductions correlate with both motor and non‐motor symptoms, suggesting similar pathophysiology, but faster disease progression, in *SNCA‐A53T* compared to sPD.^103^

[18F]FDG imaging revealed hypometabolism in caudate, frontal, parietal, and left temporal cortices in advanced‐phase *A30P‐MC*, correlating with neuropsychological deficits.^104^ In NMCs, metabolic changes are less consistent, ranging from hypometabolism in fronto‐temporal and parietal cortices as well as the caudate nucleus^98^, ^104^ to preserve glucose metabolism.^95^, ^106^

*SNCA*_*triplication*_ *MCs* are characterized, even in early disease phases, by extensive fronto‐temporo‐parietal involvement and occipital hypometabolism or hypoperfusion resembling patterns observed in dementia with Lewy bodies (DLB), supporting the aggressive and multisystem non‐motor burden of SNCA‐PD.^12^, ^15^, ^95^, ^101^, ^106^, ^110^, ^111^ Additionally, PDRP, involving hypermetabolism of pallidum, thalamus, pons, cerebellum and hypometabolism in premotor and posterior parietal cortices, has been identified in *SNCA A53T‐MC*.^98^

Finally, emerging evidence suggests increased tau deposition in the thalamus, putamen, pedunculopontine nucleus, ventral striatum, and occipital cortex in *SNCA*‐*NMCs* compared to controls.^112^

For a summary of molecular imaging findings, see Figure 1A, Table 1, S1 and S2.

**TABLE 1:** Summary of molecular imaging findings across symptomatic and asymptomatic carriers of Parkinson's disease‐related mutations

Gene	Dopaminergic system	Extra‐dopaminergic systems	Glucose metabolism/Perfusion	Brain pathology
Symptomatic mutation carriers				
SNCA	Presynaptic: ↓ in the bilateral striatum vs HC; often more severe vs sPD. Postsynaptic: largely preserved (mostly ns).	Serotonergic: ↓ in raphe nuclei, limbic and cortical regions (often more pronounced vs sPD).	↓ fronto‐temporo‐parietal‐occipital regions; ↑ basal ganglia, pons, thalamus, cerebellum.	N/A
PRKN	Presynaptic: ↓ in the striatum vs HC; ↓ (especially putamen/midbrain) or ns vs sPD. Postsynaptic: ↓ in striatum and cortical regions; ↑ in putamen in drug‐naïve state.	Limited evidence of serotonergic/noradrenergic involvement.	↓ frontal regions vs HC; ↑ cortical and subcortical regions vs sPD (compensatory patterns).	N/A
PINK1	Presynaptic: ↓ in striatum vs HC; ↓ or ns vs sPD (heterogeneous findings). Postsynaptic: mostly ns.	N/A	↓ inferomedial and fusiform gyri; ↑ precuneus, posterior cingulate gyrus, ventral striatum.	N/A
PARK7	Presynaptic: ↓ in striatum vs HC. Postsynaptic: ns.	N/A	↓ cerebellum; ↑ striatum vs HC.	N/A
LRRK2	Presynaptic: ↓ in striatum vs HC; mostly ns vs sPD, with heterogeneous findings (↓/↑ in some studies). Postsynaptic: largely preserved; occasional ↑ vs HC.	Serotonergic: ↓ striatal and ↑ limbic regions vs HC; mostly ns vs sPD. Cholinergic: ↑ cortical, limbic, thalamic regions vs HC and sPD.	↓ parieto‐frontal cortex; ↑ putamen and thalamus vs HC.	Tau pathology: ↑ occipital binding.
GBA	Presynaptic: ↓ in striatum vs HC; mixed findings vs sPD (↓, ns or ↑ depending on cohort and mutation severity).	Cholinergic: ↓ cortical, insular and parahippocampal regions vs sPD.	↓ occipito‐parietal cortex; ↑ lentiform nucleus/thalamus; ↓ frontal regions. Perfusion: ↓ posterior cortical regions vs HC.	N/A
Asymptomatic mutation carriers				
SNCA	Presynaptic: largely ns, occasional ↓ in striatum. Postsynaptic: mostly ns; occasional ↑ (compensatory).	Serotonergic: ↓ in subcortical regions.	FDG: mostly preserved; occasional ↓ in fronto‐temporal/parietal cortices and caudate vs HC.	Tau pathology: ↑ subcortical and occipital regions.
PRKN	Presynaptic: ↓ in striatum and midbrain. Postsynaptic: ↓ striatum.	N/A	Mostly ns.	N/A
PINK1	Presynaptic: ↓ or ns in striatum. Postsynaptic: ns.	N/A	N/A	N/A
PARK7	Presynaptic: ns. Postsynaptic: ns.	N/A	↓ occipito‐parietal cortex, thalamus and cerebellum vs HC.	N/A
LRRK2	Presynaptic: ↓ in striatum; occasional ↑ vs sPD (compensatory patterns).	Serotonergic: ↓ pons, thalamus, raphe nuclei; ↑ limbic and striatal regions vs HC. Cholinergic: ↑ cortical regions vs HC.	N/A	Neuroinflammation: ↑ substantia nigra.
GBA	Presynaptic: mostly ns; occasional ↑ in striatum.	N/A	Metabolism: ↓ SMA; Perfusion: ↓ (often ns) in occipito‐parietal cortex.	Neuroinflammation: ↑ substantia nigra, cortical and limbic regions.

*Note*: ↑ increased tracer uptake. ↓ decreased tracer uptake.

Abbreviations: HC, healthy controls; N/A, Not Available; ns, not significant; sPD, sporadic Parkinson's Disease.

#### PRKN

Coherently with the symmetric motor onset, *PRKN‐MCs* exhibit bilateral dopaminergic disruption.^113^, ^114^, ^115^, ^116^, ^117^, ^118^, ^119^, ^120^, ^121^, ^122^, ^123^ *PRKN‐MCs* typically show a caudo‐rostral gradient of degeneration, with some variability across studies, showing either greater caudate/midbrain or putaminal involvement compared to sPD.^115^, ^120^, ^124^ Despite similar motor symptomatology and a slower progression of dopaminergic damage in *PRKN‐MCs* compared to sPD, several imaging studies revealed more widespread dopaminergic degeneration in PRKN‐MCs.^114^, ^117^, ^118^, ^119^, ^125^ Longitudinal evidence corroborated the slower disease progression of PRNK‐PD by observing slower annual reduction of striatal (putamen: −0.5%, caudate: −2%) and midbrain (−0.6%) dopaminergic uptake, along with slow rate of progression of motor impairment, in *PRKN‐MCs* ^126^, ^127^ Nevertheless, this slower progression may not be mutation‐specific, as similar trajectories have been described in early‐onset sPD, suggesting that age at onset may influence disease progression.^128^

In drug‐naïve *PRKN‐MCs*, a similar pattern of tracer uptake to sPD, with a slight increase of D2 binding compared to controls, was observed. However, upon treatment initiation, RAC binding in *PRKN‐MCs* decreased below the levels of controls and sPD, suggesting greater susceptibility to receptor downregulation following exposure to dopaminergic medications.^129^

Additional evidence points to involvement of extra‐dopaminergic systems. Reduced [18F]DOPA (aromatic 1‐amino‐acid decarboxylase, AADC) uptake in midbrain and locus coeruleus was observed in *PRKN‐MCs* compared to sPD, suggesting the involvement of serotonergic and noradrenergic systems.^130^

Studies investigating the integrity of the dopaminergic system in *PRKN‐NMCs* are scarce. Some studies highlighted decreased integrity of striatal and midbrain regions in *PRKN‐NMCs* compared to controls,^131^, ^132^ while longitudinal data demonstrated a gradual decline in AADC activity despite the absence of clinical symptoms, again with slower progression compared to sPD.^132^, ^133^

Overall, metabolic imaging findings suggest relative preservation of cortical function. While striatal and cerebellar hypermetabolism has been reported, no evidence of cortical hypometabolism was detected in *PRKN‐MCs*, which may explain the lower incidence of cognitive impairment.^116^, ^123^ Notably, increased glucose metabolism was observed in the SMA, thalamus, and parietal regions in *PRKN‐MCs* along with enhanced connectivity between the striatum and SMA, potentially reflecting compensatory mechanisms.^125^ On the other hand, frontal hypometabolism in *PRKN‐MCs*, when present, seems to correlate with the entity of executive dysfunction.^133^ Notably, isolated observations in PRKN‐NMCs indicate preserved metabolic activity despite reduced [18F]DOPA uptake, supporting the presence of early compensatory change.^134^

For a summary of molecular imaging findings, see Fig. 1B, Table 1, S1 and S2.

#### PINK1

*PINK1‐MCs* exhibit comparable dopaminergic denervation to sPD, with the typical caudo‐rostral pattern of degeneration^135^, ^136^, ^137^, ^138^ but with symmetrical denervation^113^, ^117^, ^135^, ^136^ and slower rate of progression.^139^ Notably, the rate of striatal denervation does not seem to correlate with disease duration.^137^ Other studies, instead, observed greater degeneration in *PINK1‐MCs* than sPD, involving the caudate head and anterior putamen.^117^, ^140^

Findings in *PINK1‐NMCs* are controversial, with some highlighting reduced AADC uptake within the caudate and putamen, while others observed either increased or decreased DAT binding in *PINK1‐NMCs*.^131^, ^136^, ^137^, ^140^, ^141^ This might represent a compensatory mechanism preceding the incipit of nigrostriatal loss.

[18F]FDG‐PET studies observed hypometabolism in bilateral anteromedial and fusiform gyrus, along with hypermetabolism in the precuneus/posterior cingulate gyrus and ventral striatum of *PINK1‐MCs*.^142^

For a summary of molecular imaging findings, see Figure 2A, Table 1, S1 and S2.

#### PARK7

*PARK7‐MCs* exhibit dopaminergic deficits that are largely indistinguishable from those observed in sPD, with the typical caudo‐rostral gradient of degeneration, with predominant involvement of the posterior putamen.^131^, ^143^, ^144^ Additional findings observed decreased AADC availability and presynaptic binding, particularly in the contralateral hemisphere.^145^, ^146^ Conversely, *PARK7‐NMCs* were characterized by preserved integrity of all striatal regions.^131^, ^144^, ^146^

In *PARK7‐MCs*, hypometabolism was observed within cerebellar, thalamic and cortical regions compared to controls.^115^, ^145^, ^146^ However, a similar hypometabolic pattern was observed in sPD compared to controls.^116^, ^145^

For a summary of molecular imaging findings, see Fig. 2B, Table 1, S1 and S2.

#### LRRK2

*LRRK2‐*MCs exhibit patterns of dopaminergic degeneration that largely overlap with sPD, including the typical asymmetric and caudo‐rostral striatal denervation, with comparable severity and rate of progression.^113^, ^147^, ^148^, ^149^, ^150^, ^151^, ^152^, ^153^, ^154^, ^155^ However, contradictory evidence has emerged. For example, Simuni et al observed greater DAT striatal binding in *LRRK2*‐MCs, suggesting a slower rate of progression compared to sPD.^156^ In addition, postsynaptic dopaminergic function appears relatively preserved, as indicated by normal D2 receptor binding on RAC‐PET.^150^

Longitudinal evidence provided further insight into LRRK2‐related dopaminergic denervation. A case‐report study of an *LRRK2*‐MC observed reduced [11C]‐DTBZ (VMAT2) and [18F]FDOPA binding in the putamen at 17 years follow‐up, despite the absence of detectable dopaminergic damage at baseline.^157^ Notably, VMAT2 reduction appeared more pronounced than AADC decline, suggesting either differential sensitivity or preferential vulnerability of dopaminergic markers. The same study observed preserved integrity of the serotonergic system, as indicated by normal [11C]‐DASB binding.^157^

Imaging studies evaluating the integrity of the dopaminergic system in *LRRK2‐NMCs* are highly heterogeneous. Indeed, evidence reported increased^158^, decreased^159^, ^160^ or preserved^149^, ^152^, ^161^, ^162^, ^163^ DAT uptake in *LRRK2‐NMCs* compared to controls. Multi‐tracer studies provided further insights into LRRK2‐related variability. Reduced [11C]DTBZ (VMAT2 density) and [11C]MP (DAT) binding, but normal [18F]DOPA (AADC) uptake in *LRRK2*‐NMCs suggests that DAT downregulation could act as a compensatory mechanism, preserving AADC activity and delaying symptom onset.^150^, ^153^ This hypothesis is supported by the link between preserved AADC and increased dopamine turnover in the putamen, suggesting altered dopamine trapping or release could lead to increased dopamine turnover and, in turn, oxidative stress and cell death.^164^ Notably, the upregulation of AADC availability has also been reported in sPD patients—especially in the early phases of the disease—suggesting that such compensatory mechanisms are not exclusive to *LRRK2* mutation carriers.^165^ Nevertheless, other studies report reduced DAT and AADC uptake in LRRK2‐NMCs, reinforcing the variability of findings, which may depend on mutation type, disease stage, and cohort characteristics.^156^, ^166^, ^167^, ^168^, ^169^, ^170^, ^171^

Longitudinal multitracer PET studies provided insights into the vulnerability of dopaminergic markers in *LRRK2*‐*NMCs*. DAT binding reductions ([11C]MP) appear to represent the earliest detectable marker of subclinical dysfunction. These changes are followed by a progressive loss of [11C]DTBZ (VMAT2) binding and, later, [18F]FDOPA (dopamine synthesis) uptake. Notably, the onset of parkinsonian motor symptoms is predicted by dopamine synthesis decline.^172^ In line with these findings, lower baseline DAT uptake was identified as the most sensitive, age‐dependent marker underlying pathological processes, as LRRK2‐NMCs converting to PD within 4 years show significantly reduced binding compared to non‐converters.^170^, ^171^, ^173^

Beyond dopaminergic dysfunction, alterations in serotonergic and cholinergic pathways have been described. Increases in SERT binding in *LRRK2*‐NMCs suggest that compensatory sprouting of serotonergic terminals may boost AADC availability, preserving dopamine synthesis and delaying symptom onset.^153^ Further evaluation revealed the existence of a serotonergic spatial covariance pattern (SPDRP), correlating with disease duration, characterized by decreased tracer binding in caudate, putamen, and SN and increased uptake within the hypothalamus and hippocampus. No differences were highlighted between *LRRK2‐MCs* and sPD in SPDRP expression. Interestingly, *LRRK2‐NMCs*—without evidence of dopaminergic damage—displayed a specific pattern of serotonergic degeneration, not exhibited by sPD nor *LRRK2‐MCs*.^174^ This could either provide greater insights into disease mechanisms or identify early compensatory changes or risk factors.^174^ Finally, acetylcholinesterase activity was reported to be higher in *LRRK2*‐MCs compared to sPD, denoting the preservation of the cholinergic system, consistent with the benign PD phenotype associated with *LRRK2* mutations and the low prevalence of cognitive decline.^175^

Metabolic imaging findings also point toward a less aggressive pathological process. *LRRK2‐MCs* displayed relatively reduced metabolism in fronto‐parietal regions and the caudate, along with hypermetabolism within the SN.^151^, ^176^, ^177^ Accordingly, *LRRK2‐MCs* displayed increased connectivity within PDRP and PCRP, reflecting either milder functional pathology or compensatory mechanisms.^178^ Limited evidence also suggests the presence of tau deposits in the occipital lobe of patients with greater cognitive impairment.^151^

Finally, neuroinflammatory processes may play an early role in LRRK2‐associated disease. Increased microglial activation in the substantia nigra has been observed in LRRK2‐NMCs. *LRRK2*‐*NMCs* displayed a bilateral increase in microglial activation in the SN, supporting its potential as an early marker of underlying pathological mechanisms.^166^ Further studies should explore the contribution of microglia response and its ability to predict the conversion to PD.

For a summary of molecular imaging findings, see Figure 3A, Table 1, S1 and S2.

#### GBA1

The investigation of dopaminergic integrity in *GBA*‐*MCs* produced contrasting results, even though a consistent trend toward a faster, more severe and widespread neurodegenerative process emerges across studies.^154^, ^179^, ^180^, ^181^, ^182^ Notably, Caminiti et al observed greater dopaminergic degeneration in *GBA*‐*MCs* compared to early‐onset sPD, which is characterized by an overall benign disease course,^183^, ^184^ challenging the assumption of more severe dopaminergic damage in *GBA‐MCs*. Similarly, Simuni et al. observed higher contralateral caudate and putamen binding, suggesting lower levels of degeneration, in *GBA*‐*MCs*.^156^ However, to our knowledge, these findings were not later replicated.

Nevertheless, evidence highlighted comparable striatal denervation between *GBA‐MCs* and sPD, despite the greater motor impairment of the former, which might be explained by a negative effect of *GBA1* mutations on the subject's ability to cope with PD‐related pathology.^185^, ^186^ However, further studies are needed to confirm this association.

Additional factors may further affect disease heterogeneity. For instance, sex‐related differences have been described in GBA‐MCs, with females showing greater dopaminergic disruption despite milder motor impairment and lower risk of cognitive decline, potentially reflecting the neuroprotective and anti‐inflammatory effects of estrogens, which are abundant in dopaminergic regions.^187^

Beyond the dopaminergic system, GBA‐PD is characterized by early and severe involvement of non‐dopaminergic pathways, potentially explaining the greater vulnerability to non‐motor symptomatology of *GBA‐MCs*.^188^ Reduced vesicular acetylcholine transporter binding in temporo‐parietal cortices has been reported even in the absence of overt cognitive impairment.^189^ Consistently, metabolic imaging studies revealed increased expression of PDRP and PCRP in *GBA‐MCs* compared to sPD, suggesting greater underlying disease activity.^178^ Overall, these findings are coherent with the severe clinical phenotype of GBA‐PD.

Many studies on *GBA*‐*NMCs* revealed the absence of DAT and AADC uptake reductions compared to controls, even after a 9‐year follow‐up.^156^, ^167^, ^190^, ^191^ Conversely, mild reductions in DAT and VMAT2 binding have also been described, indicating the presence of early subclinical alterations.^192^, ^193^ In some cases, increased DAT binding has been observed despite mild clinical motor and cognitive impairment, supporting the hypothesis of early compensatory mechanisms delaying symptom onset.^161^

A distinguishing feature of *GBA‐PD* is its marked association with cognitive decline, which is reflected by severe cortical involvement. Indeed, multiple studies have consistently identified a DLB‐like occipito‐parietal hypometabolic and hypoperfusion profile, also observed in Gaucher disease patients, reinforcing the link between GBA mutations and widespread cortical involvement.^194^ Saunders‐Pullman and colleagues, instead, identified hypermetabolism in the lentiform nucleus‐thalamus of *GBA‐MCs*.^185^ Importantly, the extent of these cortical changes appears to depend on mutation severity, with “severe” GBA variants showing broader temporo‐parietal involvement, while “mild” variants exhibit patterns closer to sPD.^47^ Notably, hypometabolism in the SMA, was observed in both *GBA*‐*MCs* and *NMCs* subjects, proposing [18F]FDG as promising tool to assess prodromal brain alteration in *GBA‐PD*.^195^

Increased neuroinflammation in cortical and subcortical regions ([11C]PK11195) was linked to a higher risk of dementia.^196^ Of note, all *GBA*‐*PD* subjects (*n* = 5) included were classified as high dementia risk.^196^ Authors highlighted a negative correlation between microglial activation and cognitive performance, suggesting a contribution of neuroinflammation in the development of PD dementia.^196^ Furthermore, similar inflammatory patterns have been observed in *GBA‐NMCs* and Gaucher disease patients, involving the substantia nigra, mesencephalon, hippocampus, and occipito‐temporal cortex, indicating that neuroinflammatory processes may precede clinical manifestation and contribute to disease progression.^190^

For a summary of molecular imaging findings, see Fig. 3B, Table 1, and Supplementary Table S1–2.

## Summary and Future Perspectives

Nuclear imaging provides critical insights into neural activity and neurotransmitter alterations in genetic PD, supporting subtype classification and personalized therapeutic strategies. Given the genetic heterogeneity in PD, differential patterns of neurodegeneration observed in genetic mutations such as *PRKN*, *SNCA*, *LRRK2*, and *GBA* highlight the potential of nuclear imaging to identify biologically distinct subtypes and guide personalized medicine.

Dopaminergic imaging is instrumental in evaluating nigrostriatal integrity and revealed some mutation‐specific dopaminergic loss patterns correlating with clinical phenotype and disease progression. For instance, *PRKN* mutations are associated with slower progression of presynaptic dopaminergic dysfunction compared to sPD, aligning with milder clinical presentation, although this might relate to the early age of onset of *PRKN‐PD* rather than the genetic mutation itself, or by an interaction between the two. In contrast, *GBA‐PD* is characterized by widespread neurodegeneration, including early and severe parieto‐occipital cholinergic deficits, resembling patterns seen in DLB, and corresponding to more aggressive disease progression, increased cognitive and neuropsychiatric complications. Conversely, *LRRK2‐PD* demonstrates slower motor progression and higher survival rates, reflected by relatively preserved dopaminergic and cholinergic systems.

Nuclear imaging holds promise as a predictive biomarker in asymptomatic cohorts. Among molecular neuroimaging tools, [18F]FDG‐PET is especially sensitive to subtle neuronal and network‐level dysfunction, as it reflects regional synaptic activity and can identify disease‐specific metabolic patterns even in preclinical or prodromal stages. Evidence from other neurodegenerative disorders, such as Alzheimer's disease, indicates that [18F]FDG‐PET provides greater prognostic information than structural magnetic resonance imaging (MRI) and can reveal disease‐related changes before overt atrophy becomes measurable.^197^, ^198^

A similar framework appears to apply to genetic PD. In *GBA‐PD*, molecular imaging studies consistently report parieto‐occipital hypometabolism, while structural MRI findings are more heterogeneous, with some studies showing no significant gray matter differences in early disease stages. Longitudinal MRI investigations, however, demonstrate progressive cortical thinning—particularly in posterior regions—indicating that structural changes emerge and evolve over time.^199^ Together, these observations suggest a relative dissociation between early metabolic dysfunction and later structural alterations.

Extending this interpretation across genetic subtypes, fronto‐parietal metabolic patterns in *LRRK2‐PD* and frontal alterations in PRKN carriers may similarly reflect early network dysfunction rather than established structural damage. This is particularly relevant in NMCs, where molecular imaging provides a unique window into the presymptomatic phase of PD. Indeed, PET and SPECT studies have demonstrated that neurochemical and metabolic alterations can be detected long before the onset of motor symptoms. For example, reduced [18F]DOPA uptake in *PRKN‐NMCs* and *PINK1‐NMCs* suggests subclinical nigrostriatal dysfunction despite the absence of overt clinical manifestations, whereas preserved striatal binding in *SNCA‐NMCs* highlights variable penetrance and the likely contribution of additional modifiers, including environmental factors.

Within this framework, the identification of patient‐specific imaging patterns may improve prognostic accuracy, guide treatment decisions, and support treatment monitoring. For instance, the relative preservation of dopaminergic markers in *LRRK2‐PD* may support dopamine‐targeted therapies, which may be less effective in more aggressive forms such as *GBA‐PD*, characterized by earlier and more widespread cortical involvement. In parallel, [18F]FDG‐PET studies have shown that posterior parieto‐occipital hypometabolism can identify PD patients at higher risk of conversion to dementia with high accuracy, suggesting that metabolic imaging may help recognize individuals less likely to benefit from dopaminergic treatment escalation (LEDD increase). This metabolic pattern, resembling that observed in dementia with Lewy bodies, likely reflects prominent cholinergic denervation and may therefore aid in identifying patients who could benefit from early cholinergic intervention.^84^

Beyond individual patient management, mutation‐specific imaging signatures have important implications for clinical trials, enabling the recruitment of biologically homogeneous cohorts and reducing disease heterogeneity. In addition, identifying preferential pathways of brain vulnerability in at‐risk individuals may facilitate the detection of subjects with a higher likelihood of phenoconversion, thus supporting targeted enrollment in preclinical and prodromal studies.

Future directions should focus on integrating nuclear imaging with genetic and biochemical data to better understand neuroinflammation, proteinopathies (eg, α‐synuclein, tau), and non‐dopaminergic systems. Additionally, longitudinal studies in NMCs will be essential to establish early neuroimaging biomarkers of neurodegeneration, paving the way for preventive interventions. The main limitation of this review is the small number of participants enrolled within the genetic cohorts of the studies considered, several of which were case‐report studies. For this reason, a large, multicenter, genetic cohort will be essential to validate these imaging biomarkers and develop innovative interventions, such as gene therapy.

## Author Roles

(1) Research Project: A. Conception, B. Supervision, C. Execution; (2) Manuscript preparation: A. Writing of the first draft, B. Review and critique, C. Visualization.

CM: 1C, 2A; LG: 1C, 2A; AS: 2B, 2C;

EMV: 2B; AM: 2B;

SPC: 1A, 1B, 2B.

## Disclosures

**Ethical Compliance Statement:** Since this article is a systematic review, no patient data were collected. Thus, informed consent and IRB approval was not necessary. The authors confirm that they have read the Journal's position on issues involved in ethical publication and affirm that this work is consistent with those guidelines.

**Funding Sources and Conflicts of Interest:** This study is supported by the Italian Ministry of Health (Ricerca Corrente 2025–2027—IRCCS Mondino Foundation, Pavia).

**Financial Disclosures for Previous 12 Months:** SPC and MA are supported by the Italian Ministry of Health (grant RF‐2018‐12,366,209). SPC was supported by #NEXTGENERATIONEU (NGEU) funded by the Ministry of University and Research (MIUR), NRRP project MNESYS (PE0000006), and she is supported by the Ministry of University and Research (FIS2‐FIS‐2023‐00979). AS was supported by the Belgian National Fund for Scientific Research (grant number 40001328).

## Declarations

Authors have no relevant financial or non‐financial interests to disclose.

## Supporting information


**Table S1.** Molecular imaging finding in symptomatic mutations carriers
**Table S2.** Molecular imaging findings in asymptomatic mutations carriers

## Data Availability

The data that support the findings of this study are available from the corresponding author upon reasonable request.
