# Supplementary material for: Tracking Genetic Parkinson's Disease with Molecular Imaging: A Systematic Review
Source: Mov Disord Clin Pract. 2026 Jun 4;13(10):2379–94. doi: 10.1002/mdc3.70687 (PMC13339115; doi:10.1002/mdc3.70687)
Supplement: Supplementary file 1 [file MDC3-13-2379-s001.docx]

Supplementary Materials

Meneghini et al.

**Supplementary Table 1 –** Molecular imaging finding in symptomatic mutations carriers

| **Study** | **Imaging** | **Radioligand** | **Condition** | **Results** | | |
| --- | --- | --- | --- | --- | --- | --- |
| ***Proxies of neurotransmission and brain activity*** | | | | | | |
| Ahn et al. 2008 | SPECT | [123I]-FP-CIT | SNCA-PD vs HC | ↓ | contralateral striatum | |
| Albanese et al. 2005 | SPECT | [123I]-FP-CIT | PINK1-PD vs HC | ↓ | bilateral striatal regions | |
| Antonini et al. 2002 | SPECT | [11C]FECIT | PRKN-PD vs HC | ↓ | caudate and putamen | |
|  | SPECT | [11C]FECIT | PRKN-PD vs sPD | ↓ | putamen | |
| Barrett et al. 2013 | PET | [18F]-DOPA | GBA-PD vs HC | ↓ | bilateral striatal regions | |
|  | PET | [18F]-FDG | GBA-PD vs HC | ↓ | parietal, temporal, occipital regions | |
| Broussolle et al. 2000 | PET | [18F]-DOPA | PRKN-PD vs HC | ↓ | bilateral caudate and putamen | |
| Caminiti et al. 2022 | SPECT | [123I]-FP-CIT | GBA-PD vs sPD | ↓ | putamen, amygdala, ventral striatum, globus pallidus and hippocampus | |
| Caminiti et al. 2025 | SPECT | [18F]-FP-CIT | GBA-PD vs sPD | ↓ | putamen | |
| Chung et al. 2021 | SPECT | [18F]-FP-CIT | GBA-PD vs sPD | ns | striatal regions | |
| Cilia et al. 2015 | SPECT | [123I]-FP-CIT | GBA-PD vs sPD | ↓ | striatum | |
|  | SPECT | [123I]-FP-CIT | mild GBA-PD vs sPD | ns | striatum | |
|  | SPECT | [123I]-FP-CIT | severe GBA-PD vs sPD | ↓ | striatum | |
|  | SPECT | [123I]-FP-CIT | severe GBA-PD vs mild GBA-PD | ↓ | striatum | |
|  | SPECT | 99m-Tc-ECD | GBA-PD vs sPD | ↓ | occipital, parietal regions | |
|  | SPECT | 99m-Tc-ECD | severe GBA-PD vs mild GBA-PD | ↓ | parietal regions | |
|  | SPECT | 99m-Tc-ECD | mild GBA-PD vs sPD | ns |  | |
|  | SPECT | 99m-Tc-ECD | severe GBA-PD vs sPD | ↓ | occipital, parietal regions | |
| De Rosa et al. 2009 | PET | [18F]-FDG | LRRK2-PD vs HC | ↓ | fronto-parietal regions | |
| De Rosa et al. 2018 | PET | [18F]-FDG | LRRK2-PD vs HC | ↓ | parietal cortex | |
| Dekker et al. 2003 | PET | [18F]-DOPA | PARK7-PD vs HC | ↓ | caudate and putamen | |
|  | SPECT | [123I]-FP-CIT | PARK7-PD vs HC | ↓ | caudate and putamen | |
|  | SPECT | [123I]-IBZM | PARK7-PD vs HC | ns | caudate and putamen | |
|  | PET | [18F]-FDG | PARK7-PD vs HC | ↓ | cerebellum | |
| Dekker et al. 2004 | PET | [18F]-DOPA | PARK7-PD vs HC | ↓ | caudate and putamen | |
|  | PET | [18F]-FDG | PARK7-PD vs HC | ↑ | caudate and putamen | |
| Doostzadeh et al. 2007 | PET | [18F]-DOPA | PINK1-PD vs HC | ↓ | caudate and putamen | |
| Eggers et al. 2010 | PET | [18F]-DOPA | PINK1-PD vs HC | ↓ | caudate and putamen | |
| Ferese et al. 2015 | SPECT | [123I]-FP-CIT | SNCA-MC vs HC | ↓ | bilateral putamen | |
|  | PET | [18F]-FDG | SNCA-MC vs HC | ↓ | frontal, temporal, parietal cortices | |
| Fu et al. 2018 | PET | [11C]-DASB | LRRK2-PD vs HC | ↓ | caudate, putamen and SN |  |
|  | PET | [11C]-DASB | LRRK2-PD vs HC | ↑ | hypothalamus and hippocampus | |
|  | PET | [11C]-DASB | LRRK2-PD vs sPD | ns | caudate, putamen, SN, hypothalamus and hippocampus | |
| Grisanti et al. 2023 | SPECT | [123I]-FP-CIT | GBA-PD vs sPD | ↓ | most affected putamen and left caudate | |
| Gruel et al. 2020 | PET | [18F]-FDG | GBA-PD | ↓ | parietal and occipital cortices | |
| Guo et al. 2010 | PET | [11C]-CFT | PRKN-PD vs HC | ↓ | caudate and putamen | |
|  | PET | [11C]-CFT | PINK1-PD vs HC | ↓ | caudate and putamen | |
|  | PET | [11C]-CFT | PARK7-PD vs HC | ↓ | caudate and putamen | |
| Jensen et al. 2022 | SPECT | [123I]-FP-CIT | PRKN-PD vs HC | ↓ | striatum | |
| Hasegawa et al. 2009 | PET | [18F]-DOPA | LRRK2-PD vs HC | ↓ | caudate and putamen | |
|  | PET | [11C]-MP | LRRK2-PD vs HC | ↓ | caudate and putamen | |
|  | PET | [11C]-DTBZ | LRRK2-PD vs HC | ↓ | caudate and putamen | |
|  | PET | [11C]RAC | LRRK2-PD vs HC | ↑ | caudate and putamen | |
|  | PET | [18F]-DOPA | LRRK2-PD vs sPD | ns | caudate and putamen | |
|  | PET | [11C]-MP | LRRK2-PD vs sPD | ns | caudate and putamen | |
|  | PET | [11C]-DTBZ | LRRK2-PD vs sPD | ns | caudate and putamen | |
|  | PET | [11C]RAC | LRRK2-PD vs sPD | ns | caudate and putamen | |
| Hering et al. 2004 | PET | [123I]-FP-CIT | PARK7-PD vs HC | ↓ | bilateral striatum | |
| Hernandez et al. 2005 | PET | [18F]-DOPA | LRRK2-PD vs HC | ↓ | caudate and putamen | |
| Hilker et al. 2001 | PET | [18F]-DOPA | PRKN-PD vs HC | ↓ | striatal regions | |
|  | PET | [18F]-DOPA | PRKN-PD vs sPD | ↓ | putamen | |
|  | PET | [11C]RAC | PRKN-PD vs HC | ↓ | caudate and putamen | |
|  | PET | [11C]RAC | PRKN-PD vs sPD | ↓ | caudate and putamen | |
| Hu et al. 2006 | PET | [18F]-DOPA | PRKN-PD vs sPD | ↓ | caudate and putamen | |
|  | PET | [18F]-DOPA | PRKN-PD vs HC | ↓ | caudate and putamen | |
| Ichinose et al. 2019 | SPECT | [123I]-IMP | GBA-PD vs HC | ↓ | occipital lobe | |
| Isaias et al. 2006 | SPECT | [123I]-FP-CIT | LRRK2-PD vs sPD | ns | caudate and putamen | |
|  | SPECT | [123I]-FP-CIT | LRRK2-PD vs HC | ↓ | striatal regions | |
| Ishiguro et al. 2024 | SPECT | [123I]-FP-CIT | PARK7-PD vs HC | ↓ | striatal regions | |
| Kessler et al. 2005 | SPECT | [123I]-FP-CIT | PINK1-PD vs HC | ↓ | bilateral putamen | |
|  | SPECT | [123I]-IBZM | PINK1-PD vs HC | ns | striatal regions | |
| Khan et al. 2002 | PET | [18F]-DOPA | PRKN-PD vs sPD | ns | putamen | |
| Khan et al. 2002 | PET | [18F]-DOPA | PINK1-PD vs sPD | ↓ | anterior putamen and caudate | |
| Khan et al. 2005 | PET | [18F]-DOPA | LRRK2-PD vs HC | ↓ | caudate and putamen | |
| Kim et al. 2023 | PET | [18F]-FP-CIT | GBA-PD vs sPD | ↓ | parietal and occipital cortices | |
| Kobayashi et al. 2008 | PET | [18F]-FDG | PRKN-PD vs HC | ↓ | frontal regions | |
| Kono et al. 2010 | PET | [18F]-FDG | GBA-PD vs HC | ↓ | frontal cortex | |
| Koros et al. 2018 | SPECT | [123I]-FP-CIT | SNCA-PD vs sPD | ↓ | caudate | |
| Kruger et al 2001 | PET | [18F]-DOPA | SNCA-MC vs HC | ↓ | striatum | |
|  | PET | [ 11C]-RAC | SNCA-MC vs HC | ns | caudate | |
|  | PET | [18F]-FDG | SNCA-MC vs HC | ↓ | striatal regions, temporal and medial frontal cortices | |
| Lee et al. 2021 | PET | [123I]-FP-CIT | LRRK2-PD vs sPD | ↓ | less affected caudate and putamen | |
|  | PET | [123I]-FP-CIT | GBA-PD vs sPD | ↓ | less affected caudate | |
| Lin et al. 2008 | PET | [18F]-DOPA | LRRK2-PD vs HC | ↓ | bilateral putamen and ipsilateral caudate | |
| Liu et al. 2018 | PET | [11C]PMP | LRRK2-PD vs HC | ↑ | cortex, DMN regions and limbic regions | |
|  | PET | [11C]PMP | LRRK2-PD vs sPD | ↑ | cortex, DMN regions and thalamus | |
| Liu et al. 2023 | PET | [11C]-CFT | PRKN-PD vs sPD | ↓ | ipsilateral putamen | |
|  | PET | [18F]-FDG | PRKN-PD vs sPD | ↑ | SMA, substantia nigra, thalamus, cuneus, lingual gyrus, inferior parietal lobule, postcentral gyrus and cerebellum | |
| Martikainen et al. 2015 | SPECT | [123I]-FP-CIT | SNCA-MC vs HC | ↓ | bilateral posterior putamen | |
| McNeill et al. 2013 | SPECT | [123I]-FP-CIT | PRKN-PD vs HC | ↓ | bilateral caudate and putamen | |
|  | SPECT | [123I]-FP-CIT | PRKN-PD vs HC | ↓ | bilateral caudate and putamen | |
|  | SPECT | [123I]-FP-CIT | PINK1-PD vs HC | ↓ | bilateral caudate and putamen | |
|  | SPECT | [123I]-FP-CIT | LRRK2-PD vs HC | ↓ | caudate and putamen | |
|  | SPECT | [123I]-FP-CIT | GBA-PD vs HC | ↓ | right caudate and putamen | |
| Nishioka et al. 2006 | SPECT | [123I]-IMP | SNCA-MC vs sPD | ↓ | frontotemporal, medial-occipital lobes | |
|  | SPECT | 99m-Tc-ECD | SNCA-MC vs sPD | ↓ | frontotemporal lobes | |
| Nishioka et al. 2009 | PET | [11C]-CFT | SNCA-MC vs sPD | ↓ | anterior and posterior putamen, caudate | |
|  | PET | [11C]-CFT | SNCA-MC vs SNCA-NMC | ↓ | anterior and posterior putamen, caudate | |
|  | PET | [11C]-RAC | SNCA-MC vs SNCA-NMC | ↓ | caudate | |
|  | PET | [11C]-RAC | SNCA-MC vs HC | ns | striatal regions | |
|  | PET | [18F]-FDG | SNCA-MC vs HC | ↓ | parietal (ns), temporal (ns) and occipital regions | |
| Oeda et al. 2015 | SPECT | [123I]-IMP | GBA-PD vs HC | ↓ | bilateral parietal cortex | |
| Olgiati et al. 2015 | SPECT | [123I]-FP-CIT | SNCA-MC vs HC | ↓ | caudate and putamen | |
| Panzacchi et al. 2008 | PET | [11C]FECIT | PRKN-PD vs sPD | ↓ | bilateral caudate | |
|  | PET | [11C]FECIT | PINK1-PD vs sPD | ns | striatal regions | |
| Pavese et al. 2010 | PET | [18F]-DOPA | PRKN-PD vs sPD | ↓ | midbrain | |
| Perani et al. 2006 | PET | [11C]FECIT | SNCA-PD vs HC | ↓ | basal ganglia | |
| Portman et al. 2001 | PET | [18F]-FDG | PRKN-PD vs HC | ns | striatum | |
|  | PET | [18F]-FDG | PRKN-PD vs HC | ns | cerebellum | |
|  | PET | [18F]-DOPA | PRKN-PD vs HC | ↓ | bilateral caudate and putamen | |
|  | PET | [11C]RAC | PRKN-PD vs HC | ns | caudate | |
| Puschmann et al. 2009 | SPECT | [123I]-FP-CIT | SNCA-PD vs HC | ↓ | bilateral basal ganglia | |
|  | SPECT | ^99m^Tc-HMPAO | SNCA-PD vs HC | ↓ | parietal lobe | |
| Rango et al. 2012 | PET | [18F]-FDG | PINK1-PD vs HC | ↓ | Inferomedial and fusiform gyrus | |
|  | PET | [18F]-FDG | PINK1-PD vs HC | ↑ | precuneus/posterior cingulate gyrus and ventral striatum | |
| Ricciardi et al. 2016 | SPECT | [123I]-FP-CIT | SNCA-MC | ↓ | bilateral striatum | |
| Samaranch et al. 2010 | SPECT | [123I]-FP-CIT | PINK1-PD vs HC | ↓ | striatal regions | |
| Samii et al. 1999 | PET | [18F]-DOPA | SNCA-PD vs sPD | ns | caudate and putamen | |
|  | PET | [11C]RAC | SNCA-PD vs HC | ns | caudate and putamen | |
|  | PET | [11C]RAC | SNCA-PD vs sPD | ns | caudate and putamen | |
| Saunders‐Pullman et al. 2010 | PET | [18F]-FDG | GBA-PD vs HC | ↑ | lentiform nuclei and thalamus | |
|  | PET | [18F]-DOPA | GBA-PD vs HC | ↓ | striatum | |
| Scherfler et al. 2004 | PET | [18F]-DOPA | PRKN-PD vs sPD | ↓ | caudate, midbrain | |
|  | PET | [18F]-DOPA | PRKN-PD vs HC | ↓ | caudate, putamen and midbrain | |
|  | PET | [11C]RAC | PRKN-PD vs HC | ↓ | caudate, putamen, thalamus, frontal, parietal and temporal cortices | |
|  | PET | [11C]RAC | PRKN-PD vs sPD | ↓ | striatum, thalamus, frontal, parietal and temporal cortices | |
| Scherfler et al. 2006 | PET | [18F]-DOPA | PRKN-PD (drug-naive) vs PRKN-PD (treated) | ns | putamen | |
|  | PET | [11C]RAC | PRKN-PD vs HC (drug-naïve) | ↑ | putamen | |
|  | PET | [11C]RAC | PRKN-PD vs HC (treated) | ↓ | caudate and putamen | |
| Shi et al. 2018 | PET | [11C]-CFT | PRKN-PD vs HC | ↓ | putamen | |
|  | PET | [18F]-FDG | PRKN-PD vs HC | ↑ | putamen and cerebellum | |
| Simuni et al. 2020 | PET | [123I]-FP-CIT | LRRK2-PD vs sPD | ↑ | controlateral putamen and caudate | |
|  | PET | [123I]-FP-CIT | GBA-PD vs sPD | ↑ | controlateral putamen and caudate | |
| Slingerland et al. 2024 | PET | [18F]-FEOBV | GBA-PD vs sPD | ns | Middle and Inferior Occipital Gyrus, Parahippocampal G., Inferior Temporal G., Middle and Superior Frontal G. | |
|  | PET | [18F]-FEOBV | GBA-PD vs sPD | ↓ | Occipital, Parietal, Temporal, Superior, Anterior Frontal Cortices, Insula and Parahippocampal G. | |
| Soydaş-Turan et al. 2025 | PET | [18F]-DOPA | PRKN-PD vs sPD | ↓ | caudate | |
|  | PET | [18F]-DOPA | PRKN-PD vs HC | ↓ | caudate | |
|  | PET | [18F]-DOPA | PARK7-PD vs HC | ↓ | caudate | |
|  | PET | [18F]-DOPA | PARK7-PD vs sPD | ↓ | caudate | |
|  | PET | [18F]-FDG | PRKN-PD vs HC | ↓ | mesial prefrontal, inferior parietal and temporal cortices | |
|  | PET | [18F]-FDG | PARK7-PD vs HC | ↓ | mesial prefrontal, inferior parietal and temporal cortices | |
| Sun et al. 2023 | PET | [18F]-AV-133 | LRRK2-PD vs HC | ↓ | caudate and putamen | |
|  | PET | [18F]-FDG | LRRK2-PD vs HC | ↑ | bilateral putamen and thalamus | |
| Varrone et al. 2004 | SPECT | [123I]-FP-CIT | PRKN-PD vs sPD | ↓ | bilateral caudate | |
|  | SPECT | [123I]-FP-CIT | PRKN-PD vs HC | ↓ | striatal regions | |
| Weng et al. 2007 | SPECT | 99mTc-TRODAT-1 | PINK1-PD vs HC | ↓ | bilateral striatum | |
|  | SPECT | 99mTc-TRODAT-1 | PINK1-PD vs sPD | ↓ | putamen | |
| Wile et al. | PET | [18F]-DOPA | LRRK2-PD vs HC | ↓ | putamen | |
|  | PET | [11C]-DTBZ | LRRK2-PD vs HC | ↓ | putamen | |
|  | PET | [11C]-DASB | LRRK2-PD vs HC | ns |  | |
| Wile et al. 2017 | PET | [18F]-DOPA | LRRK2-PD vs HC | ↓ | caudate and putamen | |
|  | PET | [11C]-MP | LRRK2-PD vs HC | ↓ | caudate and putamen | |
|  | PET | [18F]-DOPA | LRRK2-PD vs sPD | ns | caudate and putamen | |
|  | PET | [11C]-MP | LRRK2-PD vs sPD | ns | caudate and putamen | |
|  | PET | [11C]-DTBZ | LRRK2-PD vs HC | ↓ | caudate and putamen | |
| Wilson et al. 2019 | SPECT | [123I]-FP-CIT | SNCA-MC vs HC | ↓ | striatal regions | |
|  | SPECT | [123I]-FP-CIT | SNCA-MC vs sPD | ↓ | caudate | |
|  | PET | [11C]DASB | SNCA-MC vs HC | ↓ | hippocampus, anterior and posterior cingulate, insula, and in the frontal, temporal, occipital cortex, ventral and dorsal raphe nuclei, caudate, putamen, thalamus, hypothalamus, amygdala, and the brainstem | |
|  | PET | [11C]DASB | SNCA-MC vs sPD | ↓ | putamen, caudate, hypothalamus, and amygdala and in the insula posterior short gyrus and insula anterior long gyrus | |
| Wurster et al. 2022 | PET | [18F]-FDG | SNCA-MC vs HC | ↓ | frontal, temporoparietal, and occipital cortex,precuneus and posterior cingulate gyrus | |
|  | PET | [18F]-FDG | SNCA-MC vs HC | ↑ | basal ganglia | |
| Xiong et al. 2016 | PET | [11C]-CFT | SNCA-MC | ↓ | bilateral striatum | |
|  | PET | [18F]-FDG | SNCA-MC | ↑ | pallidum, thalamus, pons, cerebellum | |
|  | PET | [18F]-FDG | SNCA-MC | ↓ | premotor and posterior parietal cortices | |
| Yoshino et al. 2017 | PET | [18F]-DOPA | SNCA-MC vs HC | ↓ | bilateral striatum | |
|  | PET | [11C]-DTBZ | SNCA-MC vs HC | ↓ | bilateral striatum | |
|  | PET | [11C]-MP | SNCA-MC vs HC | ↓ | bilateral striatum | |
| Zarranz et al. 2004 | SPECT | [123I]-FP-CIT | SNCA-PD | ↓ | contralateral striatum | |
| ***Tau*** ***pathology*** | | | | | | |
| Sun et al. 2023 | PET | [18F]-Florzolotau | LRRK2-PD vs HC | ↑ | occipital lobe | |

Abbreviations: 2-deossi-2-[18F]fluoro-D-glucosio ([18F]-FDG); 6-[18F]fluoro-L-DOPA ([18F]-DOPA); [18F]AV-133 ([18F]-AV-133); [18F]fluoroethoxybenzovesamicol ([18F]-FEOBV); [18F]-Florzolotau ([18F]-Florzolotau); 2β-carbometossi-3β-(4-fluorofenil)tropano ([11C]-CFT); 3-amino-4-(2-dimetilaminometil-fenil)tiolbenzamide ([11C]-DASB); dihydrotetrabenazina ([11C]-DTBZ); N-3-fluoropropyl-2β-carbometossi-3β-(4-iodofenil)nortropano ([11C]FECIT); methylphenidate ([11C]-MP); N-[11C]-methylpiperidin-4-yl propionate ([11C]-PMP); raclopride ([11C]-RAC); Default Mode Network (DMN); Gyrus (G.); Healthy Controls (HC); Iodobenzamide ([123I]-IBZM); Iodoamphetamine ([123I]-IMP); Manifesting Carrier (MC); N-ω-fluoropropyl-2β-carbometossi-3β-(4-iodofenil)tropano ([123I]-FP-CIT); Not Significant (ns); Parkinson’s Disease (PD); Positron Emission Tomography (PET); Single Photon Emission Computed Tomography (SPECT); Substantia Nigra (SN); Technetium-99m-ethyl cysteinate dimer (99mTc-ECD); Technetium-99m-labeled tropane derivative (99mTc-TRODAT-1)

↑ increased tracer uptake

↓ decreased tracer uptake

**Supplementary Table 2** – Molecular imaging findings in asymptomatic mutations carriers

| **Study** | **Imaging** | **Radioligand** | **Condition** |  | **Results** |
| --- | --- | --- | --- | --- | --- |
| ***Proxies of neurotransmission and brain activity*** | | | | | |
| Aasly et al. 2010 | SPECT | [123I]-FP-CIT | LRRK2-NMC vs HC | ↓ | putamen |
| Adams et al. 2005 | PET | [11C]-DTBZ | LRRK2-NMC vs HC | ↓ | striatal regions |
|  | PET | [11C]-MP | LRRK2-NMC vs HC | ↓ | putamen |
|  | PET | [18F]-DOPA | LRRK2-NMC vs HC | ns | caudate and putamen |
| Ahn et al. 2008 | SPECT | [123I]-FP-CIT | SNCA-NMC vs HC | ns | striatal regions |
| Artzi et al. 2017 | SPECT | [123I]-FP-CIT | LRRK2-NMC vs HC | ↓ | dorsal striatum |
| Bergareche et al. 2016 | SPECT | [123I]-FP-CIT | LRRK2-NMC vs HC | ↓ | caudate and putamen |
| Dekker et al. 2004 | PET | [18F]-DOPA | PARK7-NMC vs HC | ns | caudate and putamen |
|  | PET | [18F]-FDG | PARK7-NMC vs HC | ↓ | cerebellum, thalamus and occipito-parietal cortex |
| Droby et al. 2022 | SPECT | [123I]-FP-CIT | GBA-NMC vs HC | ns | caudate and putamen |
|  | SPECT | [123I]-FP-CIT | LRRK2-NMC vs HC | ↓ | putamen |
| Eggers et al. 2010 | PET | [18F]-DOPA | PINK1-NMC vs HC | ↓ | caudate and putamen |
| Fu et al 2018 | PET | [11C]-DASB | LRRK2-NMC vs HC | ↓ | Pons, thalamus, raphe nucleus |
|  | PET | [11C]-DASB | LRRK2-NMC vs HC | ↑ | hypothalamus, amygdala, hippocampus and substantia nigra |
| Gao et al. 2020 | PET | [18F]-DOPA | PRKN-NMC vs HC | ↓ | Striatum |
|  | PET | [18F]-FDG | PRKN-NMC vs HC | ns |  |
| Gersel Stokholm et al. 2020 | PET | [18F]-DOPA | LRRK2-NMC vs HC | ↓ | putamen |
| Guo et al. 2010 | PET | [11C]-CFT | PRKN-NMC vs HC | ↓ | caudate and putamen |
|  | PET | [11C]-CFT | PINK1-NMC vs HC | ↓ | caudate and putamen |
|  | PET | [11C]-CFT | PARK7-NMC vs HC | ns | caudate and putamen |
| Ji et al. 2020 | PET | [18F]-AV133 | GBA-NMC vs HC | ↓ | bilateral caudate |
| Hering et al. 2004 | PET | [123I]-FP-CIT | PARK7-NMC vs HC | ns | striatum |
| Hilker et al. 2001 | PET | [18F]-DOPA | PRKN-NMC vs HC | ↓ | striatal regions |
|  | PET | [11C]RAC | PRKN-NMC vs HC | ↓ | caudate and putamen |
|  | PET | [11C]RAC | PRKn-NMC vs sPD | ↓ | caudate and putamen |
| Ichinose et al. 2019 | SPECT | [123I]FP-CIT | GBA-NMC vs HC | ↓ | posterior dorsal putamen |
|  | SPECT | [123I]-IMP | GBA-NMC vs HC | ns | occipital lobes |
| Kessler et al. 2005 | SPECT | [123I]-FP-CIT | PINK1-NMC vs HC | ns | striatum |
|  | SPECT | [123I]-IBZM | PINK1-NMC vs HC | ns |  |
| Khan et al. 2002 | PET | [18F]-DOPA | PRKN-NMC vs HC | ↓ | caudate and putamen |
| Khan et al. 2002 | PET | [18F]-DOPA | PINK1-NMC vs HC | ↓ | caudate and putamen |
| Khan et al. 2005 | PET | [18F]-DOPA | PRKN-NMC vs HC | ↓ | caudate, putamen, dorsal and ventral midbrain |
|  | PET | [18F]-DOPA | LRRK2-NMC vs HC | ns | caudate and putamen |
| Kono et al. 2010 | PET | [18F]-FDG | GBA-NMC vs HC | ↓ | medial frontal cortex (SMA) |
| Kruger et al. 2001 | PET | [18F]-DOPA | SNCA-NMC vs HC | ns | striatum |
|  | PET | [11C]RAC | SNCA-NMC vs HC | ns | striatum |
|  | PET | [18F]-FDG | SNCA-NMC vs HC | ↓ | frontal, temporal cortex and left caudate |
| Liu et al. 2018 | PET | [11C]PMP | LRRK2-NMC vs HC | ↑ | cortex |
| Lopez et al. 2020 | PET | [18F]-DOPA | GBA-NMC vs HC | ns | striatum |
| Mannheim et al. | PET | [11C]MP | LRRK2-NMC vs HC | ↓ | striatal regions |
|  | PET | [18F]-DOPA | LRRK2-NMC vs HC | ↓ | striatal regions |
| Mullin et al. 2021 | PET | [18F]-DOPA | GBA-NMC vs HC | ns | striatum |
| Nandhagopal et al. 2008 | PET | [11C]MP | LRRK2-NMC vs HC | ↓ | putamen |
|  | PET | [18F]-DOPA | LRRK2-NMC vs HC | ↓ | putamen |
|  | PET | [11C]DTBZ | LRRK2-NMC vs HC | ↓ | putamen |
| Nishioka et al. 2009 | PET | [11C]RAC | SNCA-NMC vs HC | ns | striatum |
|  | PET | [18F]-FDG | SNCA-NMC vs HC | ns |  |
| Perani et al. 2006 | PET | [11C]FECIT | SNCA-NMC vs HC | ↓ | ventral striatum |
|  | PET | [11C]RAC | SNCA-NMC vs HC | ↑ | caudate and putamen |
| Pont‐Sunyer et al. 2017 | SPECT | [123I]-FP-CIT | LRRK2-NMC vs HC | ↓ | striatal regions |
| Ricciardi et al. 2016 | SPECT | [123I]-FP-CIT | SNCA-NMC | ↓ | right putamen |
|  | SPECT | [123I]-FP-CIT | SNCA-NMC | ns | striatum |
| Samaranch et al. 2010 | SPECT | [123I]-FP-CIT | PINK1-NMC vs HC | ↓ | putamen |
| Sierra et al. 2013 | SPECT | [123I]-FP-CIT | LRRK2-NMC vs HC | ↓ | striatal regions |
| Simuni et al. 2020 | SPECT | [123I]-FP-CIT | LRRK2-NMC vs HC | ns | caudate, putamen, striatum |
|  | SPECT | [123I]-FP-CIT | GBA-NMC vs HC | ↑ | caudate, putamen, striatum |
| Sossi et al. 2010 | PET | [18F]-DOPA | LRRK2-NMC vs HC | ↓ | striatal regions |
|  | PET | [11C]-MP | LRRK2-NMC vs HC | ↓ | striatal regions |
|  | PET | [11C]-DTBZ | LRRK2-NMC vs HC | ↓ | striatal regions |
| Vilas et al. 2015 | SPECT | [123I]-FP-CIT | LRRK2-NMC vs HC | ↓ | striatum |
| Wile et al. 2017 | PET | [11C]-DTBZ | LRRK2-NMC vs HC | ↓ | caudate and putamen |
|  | PET | [11C]C-MP | LRRK2-NMC vs HC | ns | caudate and putamen |
|  | PET | [18F]-DOPA | LRRK2-NMC vs HC | ↓ | caudate and putamen |
|  | PET | [18F]-DOPA | LRRK2-NMC vs sPD | ↑ | caudate and putamen |
|  | PET | [11C]C-MP | LRRK2-NMC vs sPD | ↑ | caudate and putamen |
|  | PET | [11C]-DASB | LRRK2-NMC vs HC | ↑ | hypothalamus |
|  | PET | [11C]-DASB | LRKK2-NMC vs sPD | ↑ | hypothalamus and striatum |
|  | PET | [11C]-DASB | LRKK2-NMC vs LRRK2-PD | ↑ | hypothalamus and brainstem |
| Wilson et al. 2019 | SPECT | [123I]-FP-CIT | SNCA-NMC vs HC | ns | striatal regions |
|  | PET | [11C]DASB | SNCA-NMC vs HC | ↓ | ventral and dorsal raphe nuclei, caudate, putamen, thalamus, hypothalamus, amygdala, and the brainstem |
|  | PET | [11C]DASB | SNCA-NMC vs sPD | ns |  |
| Xiong et al. 2016 | PET | [11C]-CFT | SNCA-NMC | ↓ | left striatum |
|  | PET | [18F]-FDG | SNCA-NMC | ↓ | premotor and posterior parietal cortices |
| Yoshino et al. 2017 | PET | [18F]-DOPA | SNCA-NMC vs HC | ns | striatum |
|  | PET | [11C]-DTBZ | SNCA-NMC vs HC | ns | striatal regions |
|  | PET | [11C]-MP | SNCA-NMC vs HC | ns | striatum |
| Zhang et al. 2023 | SPECT | [123I]-FP-CIT | LRRK2-NMC vs HC | ↑ | caudate |
| Zarranz et al. 2004 | SPECT | [123I]-FP-CIT | SNCA-NMC vs HC | ns | striatum |
| ***Tau*** ***pathology*** | | | | | |
| Perez-Soriano et al. 2017 | PET | [11C]-PBB3 | SNCA-NMC vs HC | ↑ | thalamus, putamen, pedunculopontine nucleus, ventral striatum and occipital cortex |
| ***Proxy of neuroinflammation*** | | | | | |
| Gersel Stokholm et al. 2020 | PET | [11C-PK11195] | LRRK2-NMC vs HC | ↑ | substantia nigra |
| Mullin et al. 2021 | PET | [11C-PK11195] | GBA-NMC vs HC | ↑ | SN, occipital and temporal lobes, cerebellum, hippocampus and mesencephalon |

Abbreviations: 2-deossi-2-[18F]fluoro-D-glucosio ([18F]-FDG); 6-[18F]fluoro-L-DOPA ([18F]-DOPA); [18F]AV-133 ([18F]-AV-133); [18F]fluoroethoxybenzovesamicol ([18F]-FEOBV); [18F]-Florzolotau ([18F]-Florzolotau); 2β-carbometossi-3β-(4-fluorofenil)tropano ([11C]-CFT); 3-amino-4-(2-dimetilaminometil-fenil)tiolbenzamide ([11C]-DASB); dihydrotetrabenazina ([11C]-DTBZ); N-3-fluoropropyl-2β-carbometossi-3β-(4-iodofenil)nortropano ([11C]FECIT); methylphenidate ([11C]-MP); N-[11C]-methylpiperidin-4-yl propionate ([11C]-PMP); raclopride ([11C]-RAC); Default Mode Network (DMN); Gyrus (G.); Healthy Controls (HC); Iodobenzamide ([123I]-IBZM); Iodoamphetamine ([123I]-IMP); Manifesting Carrier (MC); N-ω-fluoropropyl-2β-carbometossi-3β-(4-iodofenil)tropano ([123I]-FP-CIT); Not Significant (ns); Parkinson’s Disease (PD); Positron Emission Tomography (PET); Single Photon Emission Computed Tomography (SPECT); Substantia Nigra (SN); Technetium-99m-ethyl cysteinate dimer (99mTc-ECD); Technetium-99m-labeled tropane derivative (99mTc-TRODAT-1)

↑ increased tracer uptake

↓ decreased tracer uptake
